# Reproducibility of measurement of myometrial invasion in endometrial carcinoma

**DOI:** 10.1007/s00428-016-2035-5

**Published:** 2016-10-27

**Authors:** Louis J. M. van der Putten, Koen van de Vijver, Carla Bartosch, Ben Davidson, Sonia Gatius, Xavier Matias-Guiu, W. Glenn McCluggage, Gemma Toledo, Anneke A. M. van der Wurff, Johanna M. A. Pijnenborg, Leon F. A. G. Massuger, Johan Bulten

**Affiliations:** 1 Department of Obstetrics and Gynaecology, Radboud university medical center, Geert Grooteplein 10, P.O. Box 9101, 6525 GA Nijmegen, the Netherlands; 2 Department of Pathology, Anthoni van Leeuwenhoek Hospital, Plesmanlaan 121, 1066CX Amsterdam, the Netherlands; 3 Department of Pathology, Portuguese Oncology Institute-Porto, R. Dr. António Bernardino de Almeida, 4200 Porto, Portugal; 4 Department of Pathology, Norwegian Radium Hospital, Oslo University Hospital, Ullernchausseen 70, 0379 Oslo, Norway; 5 Faculty of Medicine, University of Oslo, Oslo, Norway; 6 Department of Pathology, Hospital Universitari Arnau de Vilanova, Av. Alcalde Rovira Roure, 80, 25198 Lleida, Spain; 7 Department of Pathology, Belfast Health & Social Care Trust, Knockbracken Healthcare, Saintfield Rd, Belfast, County Antrim BT8 8BH UK; 8 Department of Pathology, MD Anderson Cancer Center, Calle de Arturo Soria, 270, 28033 Madrid, Spain; 9 Department of Pathology, St. Elisabeth Hospital, Hilvarenbeekse Weg 60, 5022 GC Tilburg, the Netherlands; 10 Department of Obstetrics and Gynecology, TweeSteden Hospital, Doctor Deelenlaan 5, 5042 AD Tilburg, the Netherlands; 11 Department of Pathology, Radboud university medical center, Geert Grooteplein 10, 6525 GA Nijmegen, the Netherlands

**Keywords:** Endometrial carcinoma, Myometrial invasion, Depth of invasion, Tumor-free distance, Inter-observer variability

## Abstract

Myometrial invasion (MI) as a percentage (%MI), categorized into <50 or ≥50 %, is an important predictor of prognosis in endometrial carcinoma. Recent studies suggest that tumor-free distance (TFD) to serosa and the absolute depth of invasion (DOI) might be stronger predictors of prognosis. Although reproducibility is important in clinical practice for patient prognostication and treatment, reproducibility of these methods for the measurement of MI is largely unknown. One or two slides from 50 patients with FIGO stage I endometrioid endometrial carcinoma were viewed by seven gynecological pathologists, who were requested to measure %MI, TFD, and DOI. We categorized %MI as <50 % (including no MI) or ≥50 %, TFD as ≤1.75 or >1.75 mm (including no MI), ≤7 or >7 mm (including no MI), and ≤10 or >10 mm (including no MI) and DOI as <4 mm (including no MI) or ≥4 mm. Light’s kappa for multi-rater agreement was calculated. The %MI, TFD, and DOI could be measured in 88, 83, and 79 % of cases, respectively. Kappa was 0.75 for %MI, 0.77, 0.73, and 0.69 respectively for TFD with cutoffs of 1.75, 7, and 10 mm, and 0.59 for DOI. Pathologists reach substantial agreement when measuring %MI and TFD and moderate agreement when measuring DOI. The %MI can be measured in more cases than TFD and DOI. This supports the use of %MI in daily clinical practice, but future studies should compare %MI and TFD more extensively, including inter-observer variability.

## Introduction

Endometrial carcinoma is the most common gynecological malignancy in developed countries, and its incidence is increasing [1, 2]. Primary treatment of endometrial carcinoma consists predominantly of hysterectomy and bilateral salpingo-oophorectomy. Additional staging is typically undertaken for non-endometrioid and high-grade endometrioid carcinomas and when tumor stage is advanced. Most patients are diagnosed with FIGO stage I disease and low-grade (grade 1 or 2) endometrioid histology and have a good prognosis [2]. After primary surgery, the decision to administer adjuvant radiotherapy to prevent locoregional recurrences relies on the presence of predictors of poor outcome, such as high tumor grade, lymphovascular invasion, deep myometrial invasion (MI), and patient age >60 years [2].

Traditionally, the percentage of myometrial invasion (%MI), categorized as <50 or ≥50 %, is one of the parameters used in the determination of the need for adjuvant radiotherapy [3–5]. However, more recently, two other methods of measuring MI have been proposed: tumor-free distance (TFD) to serosa (the distance in millimeters between the deepest point of invasion and the serosa) and absolute depth of invasion (DOI, the distance in millimeters between the endometrial/myometrial junction and the deepest point of MI). A study comparing TFD and DOI and another study comparing %MI, TFD, and DOI concluded that TFD is superior in predicting disease extension as well as outcome [6, 7]. Two comparable studies, on the other hand, have shown that DOI is superior in predicting nodal involvement, recurrent disease, and disease-related mortality [8, 9]. One study comparing TFD and DOI concluded that DOI is a stronger predictor of outcome, but TFD is easier to measure, but kappa statistics were not reported [10].

If measurement of TFD or DOI is superior to that of %MI, it might improve identification of high-risk patients and individualization of adjuvant treatment. However, reproducibility of these measurements is important to support their prognostic value in daily clinical practice. Because all previous studies were single-center studies and measurements were performed by a limited number of pathologists, reproducibility of TFD and DOI is currently unknown. Some studies have reported on reproducibility of %MI, but only one study included kappa statistics with a kappa value of 0.83 [11]. The aim of our study was to assess inter-pathologist reproducibility of %MI, TFD, and DOI.

## Materials and methods

### Included cases

Slides from patients treated for stage I endometrioid endometrial carcinoma at the Radboud university medical center (Radboudumc), Nijmegen, the Netherlands, between January 1999 and December 2009 were reviewed by a gynecological pathologist (JB).

All pathologists collaborating in the European Network for Individualized Treatment of Endometrial Cancer (ENITEC) were invited to participate in this study, and seven expressed their interest. The sample size calculation was based on previous studies assessing reproducibility of the %MI measurement, as the kappa for TFD and DOI measurements is unknown [11–14]. Based on a kappa of 0.8 for %MI, we calculated that 50 cases should be included in order to have 90 % assurance that the two-sided 95 % confidence interval would be no more than 0.1 [11, 15].

### Myometrial invasion measurement

**Fig. 1:**
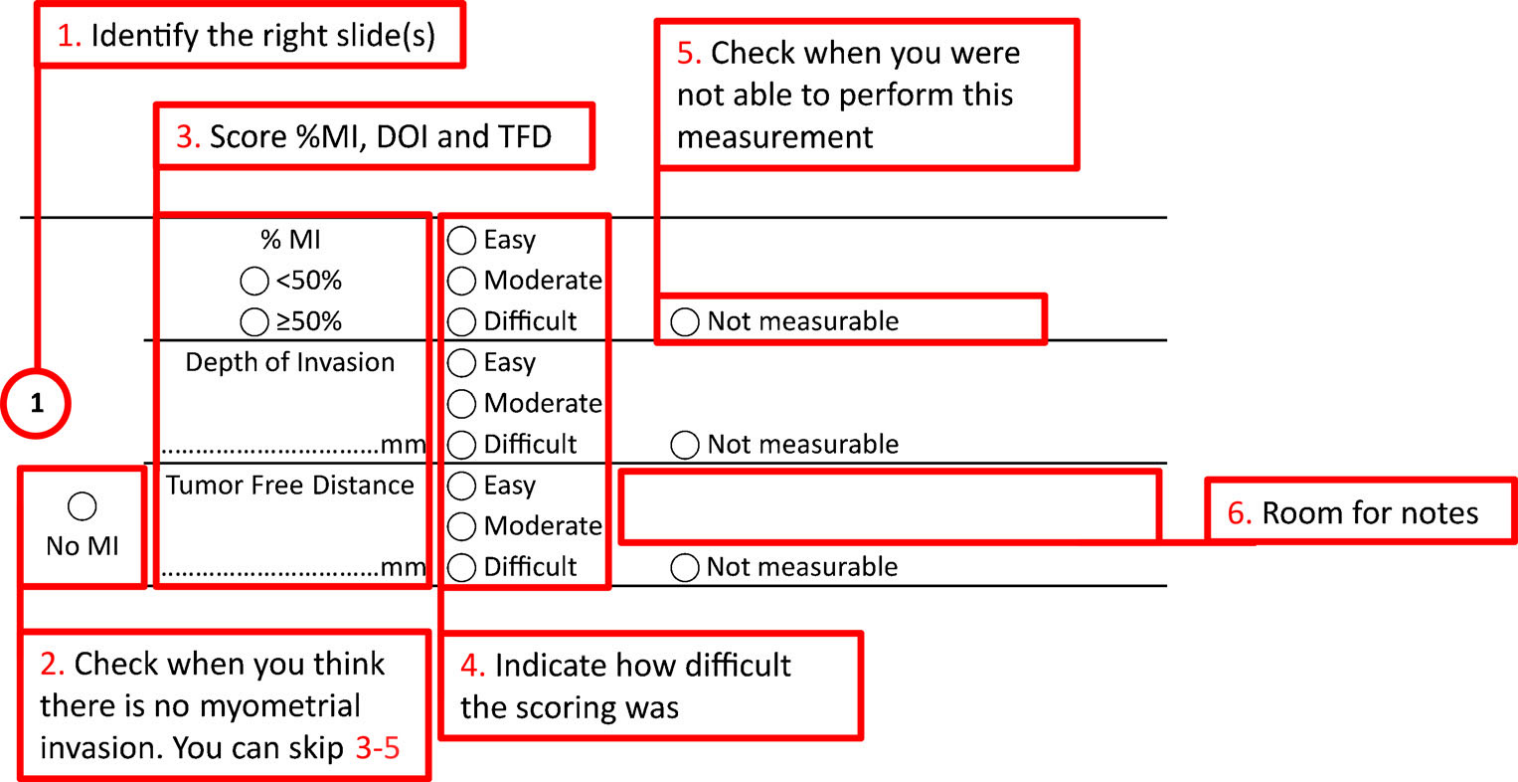
Scoring instructions

**Fig. 2:**
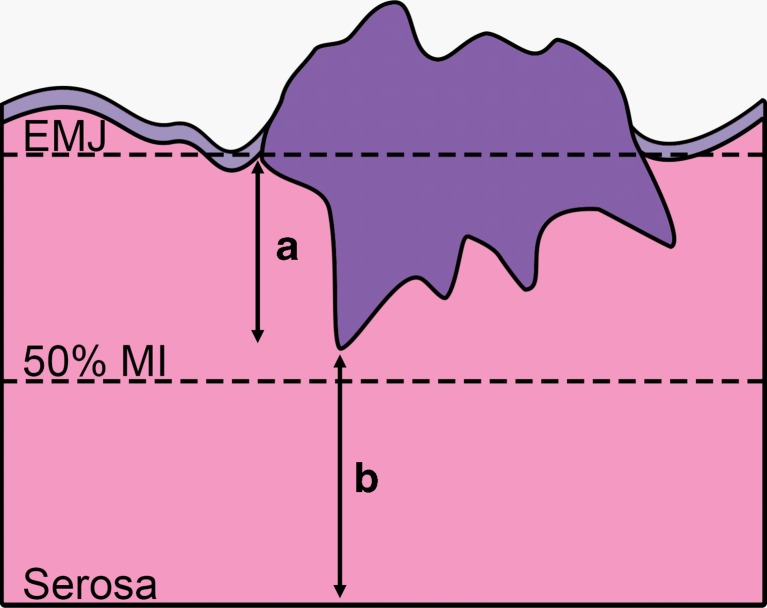
Drawing of the different measuring methods in an endometrial carcinoma with <50 % myometrial invasion. The *dotted lines* show the position of the endometrial/myometrial junction (*EMJ*), and the line where the tumor would invade half of the myometrium (*50 % MI*). The *arrows* show the absolute depth of invasion (*A*) and tumor-free distance (*B*) measurements

All cases were assessed independently by seven expert gynecological pathologists who work in large referral centers (AW, KV, CB, SG, BD, WGM, and GT), using the same set of slides. Scoring was performed according to the instructions shown in Fig. 1. For every case, the presence of MI had to be determined. In cases with MI, the three different methods for MI, as shown in Fig. 2, needed to be scored %MI (not measurable, <50 or ≥50 %), TFD (not measurable or the number of millimeters from the deepest point of invasion to the serosa), and DOI (not measurable or the number of millimeters from the endometrial/myometrial junction to the deepest point of invasion). Moreover, a perception of the difficulty of each measurement (easy, moderate, or difficult) had to be reported by each pathologist. There was also an option to provide comments after every measurement.

### Statistical analysis

For statistical analysis, %MI was categorized as <50 % (including no invasion) or ≥50 %. Reproducibility of TFD was calculated for all three previously reported cutoff values: ≤1.75 or >1.75 mm (including no invasion), ≤7 or >7 mm (including no invasion), and ≤10 or >10 mm (including no invasion) [6, 7, 9, 10]. Only one earlier study described a cutoff for DOI, which was categorized as <4 mm (including no invasion) or ≥4 mm [9].

Light’s Kappa for multi-rater agreement was calculated for categorized %MI, TFD, and DOI scores, bootstrapped (1000 runs), and 95 % confidence intervals were calculated. Missing scores were excluded in a pairwise fashion. Kappa was categorized into slight (0.01–0.20), fair (0.21–0.40), moderate (0.41–0.60) substantial (0.61–0.80), or almost perfect (0.81–0.99) agreement [16]. R statistical software was used to perform the calculations [17].

## Results

### Myometrial invasion measurement

The results of the measurements are shown in Table 1. As there were 50 cases, measured by seven pathologists, a total of 350 measurements were possible per method. In 95 % of the 350 measurements, the pathologists were able to assess whether or not there was MI, ranging from 82 to 100 % of the 50 measurements per pathologist. For the %MI, TFD, and DOI measurements, this was 88 % (64–98 %), 83 % (78–88 %), and 79 % (24–100 %), respectively. For the presence of MI and the measurement of TFD, the median number of measurements per case available to calculate Light’s multi-rater kappa was seven; for the %MI measurement, this was 6.5; and for the DOI measurement, 6. Almost all cases had two or more measurements per method, allowing calculation of a kappa value. In four cases, it was impossible to calculate the kappa value for the TFD measurement, because no or only one measurement was performed.

**Table 1:** Characteristics and reproducibility of myometrial invasion measurements

Number of cases	50
Measurements possible per method	350
Is there myometrial invasion?	
Measurable	331 (95 %, range﻿ 82-100%)
Median measurements per case	7 (range 3-7)
No myometrial invasion	79 (24 %)
Myometrial invasion	252 (76 %)
Kappa (95 % confidence interval)	0.63 (0.5–0.78)
Percentage of myometrial invasion	
Measurable	307 (88 %, range 64-98%)
Median measurements per case	6.5 (range 3-7)
Myometrial invasion <50 %	220 (72 %)
Myometrial invasion ≥50 %	87 (28 %)
Kappa (95 % confidence interval)	0.75 (0.60–0.87)
Tumor-free distance	
Measurable	291 (83 %, range 78-88%)
Median measurements per case	7 (range 0-7)
Median tumor-free distance	7 mm (range 0.8-19mm)
Tumor-free distance >1.75 mm	273 (94 %)
Tumor-free distance ≤1.75 mm	18 (6 %)
Kappa (95 % confidence interval)	0.77 (0.60–0.90)
Tumor-free distance >7 mm	181 (62 %)
Tumor-free distance ≤7 mm	110 (38 %)
Kappa (95 % confidence interval)	0.73 (0.60–0.85)
Tumor-free distance >10 mm	137 (47 %)
Tumor-free distance ≤10 mm	154 (53 %)
Kappa (95 % confidence interval)	0.69 (0.54–0.79)
Depth of invasion	
Measurable	275 (79 %, range 24-100%)
Median measurements per case	6 (range 2-7)
Median depth of invasion	5 mm (range 0.1-25 ﻿mm)
Depth of invasion <4 mm	156 (57 %)
Depth of invasion ≥4 mm	119 (43 %)
Kappa (95 % confidence interval)	0.59 (0.41–0.76)

**Fig. 3:**
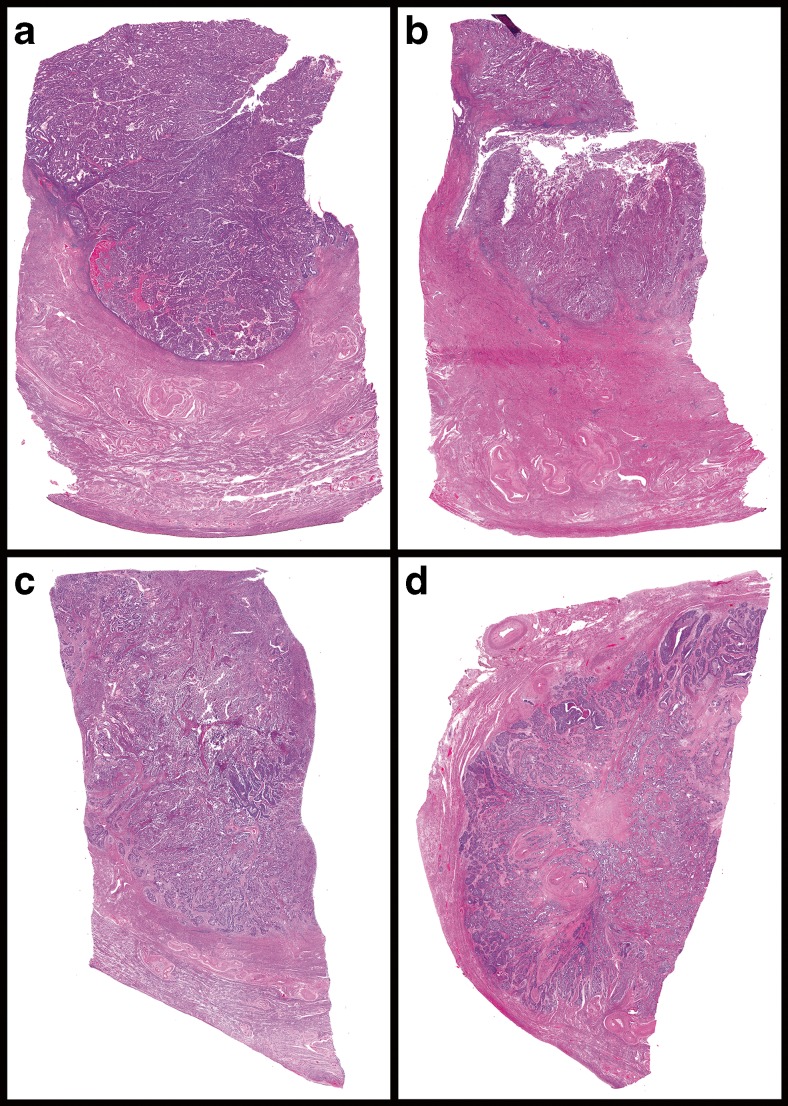
Several examples of slides scored in this study. Slides **a** and **b** were scored with little agreement concerning the DOI, and it was commented that it was hard to distinguish the endometrial/myometrial junction. Slides **c** and **d** on the other hand were scored with perfect agreement for all measurements.

The pathologists reported MI in 76 % of the measurements with a kappa of 0.63 and ≥50 % myometrial invasion in 28 % of the measurements with a kappa of 0.75. The median TFD was 7 mm (range 0.8 to 19 mm). TFD was ≤1.75 mm in 6 %, ≤7 mm in 38 %, and ≤10 mm in 53 % of the measurements with kappa values of 0.77, 0.73, and 0.69, respectively. Median DOI was 5 mm (range 0.1 to 25 mm), ≥4 mm in 43 % of the measurements with a kappa of 0.59. Examples of cases with good or poor reproducibility are shown in Fig. 3.

### Difficulties in measuring myometrial invasion

Table 2 shows how the pathologists rated the difficulty of the three measurements relative to the percentage of cases measured. For the %MI, the number of measurements performed with a reported difficulty was 211; for TFD, this was 201; and for DOI, 189. The measurements were perceived to be easy in 54 % for %MI, 72 % for TFD, and 24 % for DOI; they were moderate in 30 % for %MI, in 21 % for TFD, and in 43 % for DOI and difficult in 16 % for %MI, in 7 % for TFD, and in 33 % for DOI. For all three measurements, the kappa value of the perceived difficulty was smaller than 0.1.

**Table 2:** Difficulty of the myometrial invasion measurements of cases with myometrial invasion

	%MI	TFD	DOI
Percentage measurable	88 %	83 %	79 %
Number of measurements with MI and known difficulty	211	201	189
Percentage difficulty (range)			
Easy	54 % (21–77%)	72 % (59–93%)	24 % (0–39% )
Moderate	30 % (23–54%)	21 % (4–50%)	43 % (28–68%)
Hard	16 % (0–29%)	7 % (0–23%)	33 % (24–64%)

## Discussion

This study shows that gynecological pathologists reach substantial agreement when measuring %MI and TFD and moderate agreement when measuring DOI. Pathologists found measuring DOI more difficult than measuring %MI and TFD.

### Myometrial invasion measurement

It is widely accepted that high tumor grade, non-endometrioid histology, lymphovascular space invasion, and deep myometrial invasion are predictors of poor prognosis in endometrial carcinoma and important parameters to decide on individualized treatment [2]. Although many studies reported on reproducibility of tumor grading and histological typing, reports on reproducibility of assessment of MI and lymphovascular invasion are limited [18–21]. One study reported a Cohen’s kappa value of 0.83 for two pathologists measuring myometrial invasion in 177 cases of endometrial cancer [11]. Other studies determined the percentage of agreement between pathologists when measuring MI, but without calculating the kappa value. Ali et al. reported a discrepancy between the original %MI and the specialist reviewer %MI measurement in 12 % of endometrial cancer cases [14]. Jacques et al. reported discrepancies between MI measured by the pathologist who reported the case and a reviewing pathologist in 31.5 % of cases [12]. In that study, MI was categorized as not present, less than one third, and equal to or more than one third and discrepancies most commonly resulted in upstaging from no to less than one third MI. A comparable study by Chafe et al. described differences between the original pathology report and a review in 34 % of 226 cases, but the percentage of cases with discrepancies in the categorization of %MI was not separately mentioned [13]. Lindauer et al. assessed the prognostic value of the TFD measurement in 153 cases, but the reproducibility between two pathologists was only determined in five cases [6].

We show that gynecological pathologists reach substantial agreement with respect to the presence of MI and the measurement of %MI and TFD, and moderate agreement with respect to the measurement of DOI [16]. Interestingly, for both %MI and TFD, reproducibility was better than that for assessment of the presence of MI. This is in line with the studies of Jacques et al. and Ali et al., who found most discrepancies between cases with no MI and cases with superficial MI [12, 14]. Because the revised 2009 FIGO staging system does not differentiate between no MI and superficial MI, this finding does not affect staging and has been shown to be of no clinical significance [22].

In comparing agreement between pathologists with respect to %MI, TFD, and DOI measurements, the best agreement was reached when measuring TFD with a cutoff of 1.75 mm. This was closely followed by the %MI measurement and TFD with cutoffs of 7 and 10 mm. The most relevant cutoff for TFD needs to be determined, but the differences in reproducibility are small and probably without clinical importance, as are the differences between the reproducibility of the %MI and TFD measurements.

Measuring MI is more difficult in the presence of an irregular endometrial/myometrial junction, of a polypoid tumor, or of adenomyosis, and also when the pattern of MI is unusual, such as diffusely infiltrative, or microcystic, elongated, and fragmented (MELF) [12, 23–26]. Because these are not yet regularly reported in daily clinical practice, this was beyond the scope of our study. However, it would be interesting to analyze the effect of different invasion patterns on the reproducibility of MI measurements.

### Difficulties in measuring myometrial invasion

Pathologists found measurement of DOI more difficult than that of %MI and TFD, which is reflected in the lower average reproducibility of these measurements. However, perception of difficulty per case varied widely between pathologists, as reflected in a low kappa value.

Comments of the participating pathologists indicated that sampling and sectioning of the endometrium and myometrium varies between institutions. Nonetheless, moderate to substantial agreement was obtained. However, further standardization of the guidelines might decrease inter-observer variability of these three measurements, which might improve their prognostic value. Possible improvements might be (1) standardization of the method to open the uterus as well as the location and direction in which the tissue samples are taken relative to the tumor, the myometrium, and the serosa; (2) photographic documentation of the specimen; and (3) standardization of identification of the deepest point of invasion and the definition of the endometrial/myometrial junction.

### Strengths and weaknesses of this study

This is the largest study assessing inter-pathologist reproducibility of MI measurement and the first assessing the reproducibility of the TFD and DOI measurement. Although the 95 % confidence intervals of the kappa values were slightly wider than expected, in part due to the fact that not all measurements were performed, they remained acceptable. For a study on reproducibility in daily practice, our use of slides from daily practice rather than cases optimized for measurability makes the results relevant for daily practice. A limitation is that these slides were from one institution, while significant differences exist between institutions regarding sectioning and measuring procedures. Standardization of guidelines might further improve inter-observer reproducibility.

## Conclusions

We show that gynecological pathologists reach substantial agreement when measuring %MI and TFD, but only moderate agreement when measuring DOI. Measurement of %MI and TFD was perceived to be easier than DOI measurement and %MI was the measure most often successful. This supports the use not only of %MI but also of TFD. These two parameters merit further study, always by at least two pathologists as this will provide insight in inter-observer variability. Guidelines for gross examination, sectioning, and measuring of MI should be standardized to improve the inter-observer variability and improve on prognostic value.
